# Single stage en bloc resection of a recurrent metastatic osteosarcoma of the pediatric lumbar spine through multiple exposures – a novel approach

**DOI:** 10.1051/sicotj/2018028

**Published:** 2018-07-13

**Authors:** Saurabh Gupta, Zachary S. Stinson, Rex A. Marco, John P. Dormans

**Affiliations:** 1 Texas Children's Hospital, Baylor College of Medicine, Houston, TX USA; 2 Spine Surgery and Musculoskeletal Oncology, Houston Methodist Hospital, Houston, TX USA; 3 Paediatric Orthopaedics Department, Texas Children's Hospital, Baylor College of Medicine, Houston, TX USA

**Keywords:** Recurrent spine osteosarcoma, En bloc spondylectomy, Single stage multiple exposure paediatric spine surgery

## Abstract

To obtain a wide resection and safe margins in recurrent spine osteosarcoma, the surgical approach can include – posterior only, combined posterior and anterior, and combined posterior and anterior with a return to posterior in multiple stages. In our case, we used a novel approach of multiple extensile exposures circumferentially in a single stage with a single surgical prep. We present the case of a 9-year-old female with a history of metastatic osteosarcoma, who previously underwent an attempted en bloc resection with an L3 corpectomy and left below knee amputation. At 1 year follow-up, she developed a recurrent solitary spine lesion at the previous surgical resection site. An additional attempt at complete surgical resection was performed with a complex en bloc L2, L3, L4 corpectomy with removal of deep spinal implants and anterior and posterior spinal fusion with instrumentation and revision decompressive laminectomy. The patient had a good functional outcome without neurological deficits, except those resulting from resection of involved lumbar nerve roots. At last follow-up of 5 months, there was no local recurrence or distant metastasis. This approach for revision resection of recurrent spinal osteosarcoma can be performed successfully with clean margins in a safe manner.

## Introduction

Osteosarcoma is the most common primary skeletal malignancy encountered in children and adolescents [1]. It is an aggressive tumor derived from primitive bone forming mesenchymal cells [2]. For an optimal outcome, complete surgical excision is required. Total en bloc spondylectomy is an accepted surgical procedure for primary spine tumors [3]. However, in cases of recurrent metastatic osteosarcoma involving the spine, treatment options are more controversial due to a poor prognosis with or without surgical excision [4]. In addition, surgical options are limited and technically demanding in cases of revision and recurrence of spine osteosarcoma due to surrounding vital structures (i.e. spinal cord, nerve roots, ureter, great vessels), deranged anatomic restrictions, previous retained implants and extensive scarring [4]. To our knowledge, there are no previous reports of revision en bloc spondylectomy for recurrent metastatic osteosarcoma to the spine. To obtain a wide resection and clear margins, various studies have recommended different approaches that include – posterior only, combined posterior and anterior in a single stage or multiple stages, and combined posterior and anterior with a return to posterior in multiple stages [5]. In our case, we used a novel approach with multiple extensile circumferential exposures in a single stage without the need to break the sterile field.

## Case presentation

**Figure 1:**
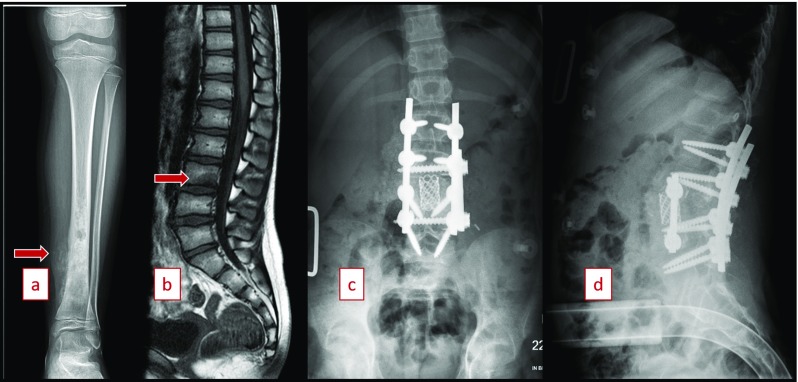
(a) AP view of primary osteosarcoma of left tibia (February 2016). (b) T1 SAG of lumbar spine with metastatic lesion involving vertebral body of L3 (February 2016). (c) PA postoperative image following initial en bloc resection with L3 corpectomy (March 2016). (d) Lateral postoperative image following initial en bloc resection with L3 corpectomy (March 2016).

In this article, we report the case of a 9-year-old female patient with no previous medical history and no significant family history of osteosarcoma or any other bone tumor, who presented in our clinic on January 2016 with a 1 year history of pain in her low back and left lower limb. She was diagnosed with osteosarcoma of the left tibia with a solitary metastasis in her L3 vertebrae on February 2016. She was subsequently treated with a left below knee amputation and L3 corpectomy with posterior spinal fusion and instrumentation from L1 to L5 with decompression laminectomy at L2-3, and L3-4 through a posterior and left thoracoabdominal approach in March 2016 (Figure 1).

**Figure 2:**
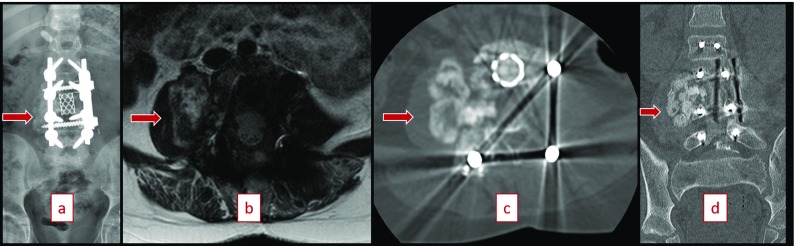
(a) PA of spine 9 months post initial resection with recurrence (November 2016). (b) T2 Axial MRI demonstrating recurrent osteosarcoma extending into right psoas muscle (December 2016). (c, d) CT scan cuts demonstrating recurrent osteosarcoma with previous retained implants (December 2016).

**Figure 3:**
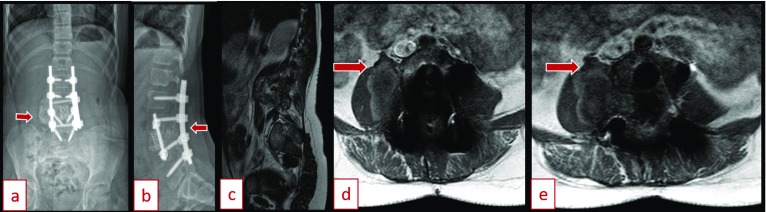
(a) EOS (Low dose radiation imaging device) spine PA view preoperative (February 2017). (b) Spine lateral view preoperative (February 2017). (c) Preoperative sagittal MRI demonstrating recurrent osteosarcoma (December 2016). (d, e) Preoperative axial MRI demonstrating recurrent osteosarcoma proximity to neurovascular structures (December 2016).

**Figure 4:**
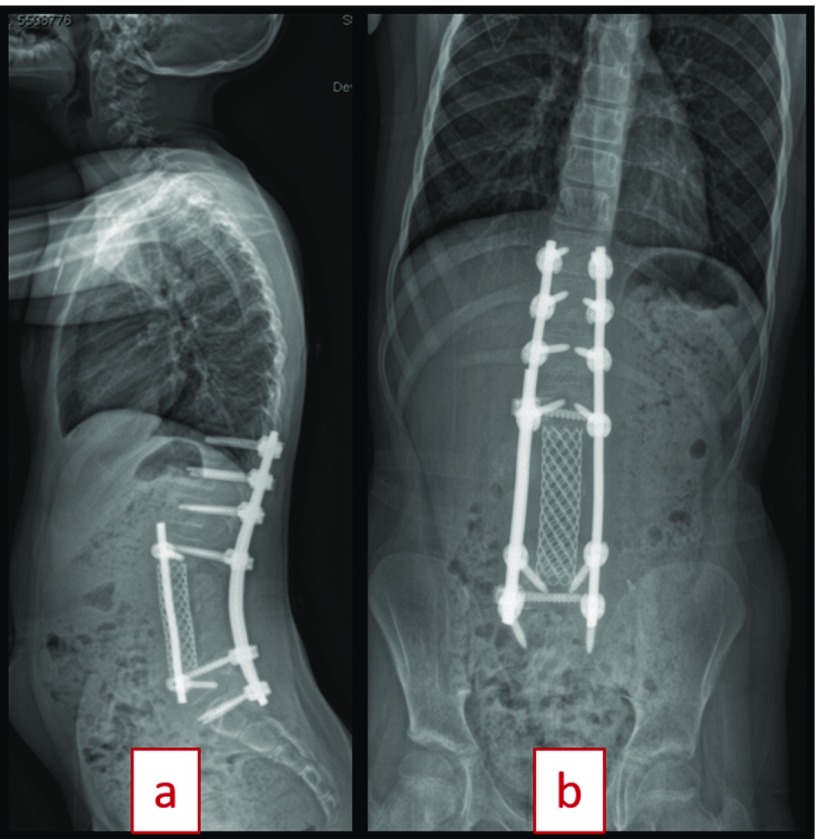
(a) Spine PA view five-month postoperative from revision en bloc resection (August 2017). (b) Spine lateral view five-month postoperative from revision en bloc resection (August 2017).

She was found to have an abnormal bone scan with a lesion at the L3 level 3 months after completing chemotherapy in November 2016. MRI scan and ultrasound guided fine-needle aspiration cytology (FNAC) of right paraspinal psoas tissue confirmed recurrent osteoblastic osteosarcoma on December 2016 (Figure 2). On physical examination, she was able to ambulate with use of a below knee prosthesis and demonstrated no neurological deficits. The patient was started on second line drugs, including two cycles of ifosfamide/etoposide. Previous implants were well in place. Imaging was performed with radiographs, CT scan, bone scan, PET scan, and MRI scan with contrast enhancement to confirm only a single metastatic site (Figure 3). For therapeutic strategy determination, the patient was introduced to our local tumor board. Preoperative workup was completed, and surgery was planned for a complex en bloc resection of L2, L3, and L4 with removal of deep spinal implants with anterior and posterior spinal fusion and instrumentation (Figure 4). The option of nonoperative palliative care was offered to the patient and her family, but they elected to proceed with en bloc resection to maximize her chances of survival, in spite of high surgical risk and an overall poor prognosis. They were informed preoperatively that a complete resection would require sacrificing her nerve roots at L2, L3 and L4. A palliative decompression was not offered for the revision procedure as a treatment option as the patient was not complaining of pain or neurological symptoms, and it would not have improved her life expectancy.

## Procedure

The procedure was performed on March 2017 with the patient under general anesthesia on a radiolucent table with a circumferential sterile prep and draping performed from cephalad to her nipple line down to her groin, with her left lower extremity also prepped in the sterile field.

In the prone position, the previous instrumentation was exposed and removed through a standard longitudinal posterior approach. Using fluoroscopy, bilateral pedicle screws were placed at T10, 11, 12, L1 and L5 on both sides. Laminectomies from L1 to L5 were then performed, and there was a tremendous amount of scarring of the dura to the neural elements. Intraoperatively, two durotomies were noted and eventually repaired, and sealed with Evicel. L1-2 and L4-5 level discectomies were performed bilaterally. Transection of the right L2, L3 and L4 nerve roots was completed, and anterior mobilization of the mass was performed. Posterior rods were placed and secured from T10 to L5, and the patient was repositioned in the same sterile field.

In the right lateral decubitus with left side up, a revision illealthoracoabdominal approach was performed through the previous healed surgical scar. The ureter was protected and the great vessels were found to be adhered to the spine with a tremendous amount of scarring. The L1-2 and L4-5 left side discs were mobilized and transection completed. Further mobilization of the mass was performed as much as possible. The right flank incision was closed and the patient repositioned.

In the left lateral decubitus with right side up, an additional thoracoabdominal approach was performed exposing the psoas and great vessels. Care was taken to mobilize the common iliac, as well as the inferior vena cava. The inferior vena cava was adhered to the recurrent tumor anteriorly at L3, the top of L4, and the bottom of L2. It was carefully dissected off the specimen. The intimacy of the great vessel with the tumor forced a very narrow margin in this location. The tumor mass involving L2, L3, and L4 was then removed en bloc with the majority of right psoas muscle. Another durotomy was noted on the right side, and it was repaired with 6-0 Prolene. Thorough pulsatile copious irrigation was performed. There was no leakage of CSF noted with Valsalva maneuver. Adjacent tissue was removed and sent for further evaluation of clear margins. Anterior interbody instrumentation was then performed with a Dupuy Synthes mesh cage and packed with crushed cancellous bone allograft in between L1 and L5.

The posterior wound was reopened for simultaneous anterior and posterior exposure while in the left lateral decubitus position. The cage was then compressed posteriorly and excellent fixation was noted. Additional spinal instrumentation was placed anteriorly from L1 to L5. Compression across this instrumentation was also performed. Thorough decortication of the facets in the posterior aspect of the spine was then performed at T10-11, T11-12, T12-L1, L1-L2, and then L5-S1. Local bone autograft and additional crushed corticocancellous allograft bone and vancomycin powder were placed. The wounds were closed in multiple layers over deep drains. The patient was brought to recovery room in stable condition.

## Outcome

Total operative time was 14 hours (posterior, right and left anterolateral) with an estimated blood loss of approximately 6 000 mL. Intraoperatively, a total of 12 units of packed red blood cells were transfused. The patient stayed at the intensive care unit for 2 days and postoperatively there were, apart from neuropathic pain and hyperalgesia both lower limbs, no further major or minor complications.

A specimen with measurements of 12.0 × 7.0 × 6.0 cm was submitted for histopathologic evaluation. This revealed osteoblastic osteosarcoma. There was no tumor involvement of the surgical margins and none of the surrounding tissue samples contained evidence of malignant tissue. The closest margin was less than 0.1 cm at the vascular groove. The pathology report demonstrated negative margins and 80% tumor necrosis.

Postoperative management included pain management and physical therapy to address significant neuropathic pain and hyperalgesia in both lower extremities. She had numbness in the L2, L3 and L4 dermatomal distributions on the right side, as well as weakness with attempted hip flexion, knee extension and ankle dorsiflexion. In spite of her motor deficits created by the transaction of her right L2, L3 and L4 nerve roots, she was quite resilient and able to ambulate with the assistance of a walker. She was ready for discharge 2 weeks postoperatively, and was not requiring pain medication at that time. She had postoperative weight loss, which was attributed to nausea and poor appetite after surgery, but this resolved and now she's gaining weight. All surgical wounds healed uneventfully. She returned for follow-up at 1 month and 5 months, respectively, and progressed well with mild numbness and weakness in her right lower extremity without evidence of tumor recurrence. She was able to ambulate without the use of walker with a below knee prosthesis to her left lower extremity and an ankle foot orthosis (AFO) to her right lower extremity.

## Discussion

This case is unique as the patient was treated surgically with a single stage with use of a posterior midline as well as, right and left anterolateral thoracoabdominal approaches for an en bloc resection of a recurrent metastatic spinal osteosarcoma, within one year of a previous attempted en bloc resection. At 5 months of follow-up, her results were satisfactory without tumor recurrence. We could not find any other published report/paper of a similar surgical technique. Such a procedure is technically demanding due to the high risks to vital structures in expanding resection margins. Also in revision resection surgery, the risk of complication is increased due to altered anatomy, extensive scarring of surrounding neurovascular structures and previous implants in situ. As per Tomita et al. and a few additional studies, a posterior approach is required for total en bloc excision and stabilization. Depending on the extent and nature of the disease, an additional anterior approach can be added [3,4,6–8]. Alternatively, a multiple stage approach has been reported [9]. In contrast to these reports, our case was performed in a single stage with three approaches, which seemed to be mandatory to obtain a circumferential resection with optimal margins in view of the extensive adhesions, scar tissue formation, tumor mass, and previous retained implants. Boriani et al. reported around a 46% complication rate in revision surgeries, and this high rate depended mainly on the number of vertebrae and the extent of soft tissue resected [7].

##  Conclusion and clinical message

In an extremely difficult case of recurrent spine osteosarcoma, we have demonstrated a single stage en bloc resection and stabilization through multiple exposures with clean margins. Adequate preoperative evaluation with use of a, multi-disciplinary team at a tertiary spine care center with the involvement of a highly experienced expert surgical team were essential to the successful outcome of this management approach. At the present time, there is no evidence of recurrent tumor, and the patient is active and progressing well at 5 months of follow-up. Even though the reported follow-up is very short, her outcome is very encouraging and can be considered successful in the setting of what otherwise is a fatal disease.

## Conflict of interest

The authors declare that they have no conflict of interest.
